# Dechlorination of polychlorobiphenyl degradation metabolites by a recombinant glutathione *S*‐transferase from *Acidovorax* sp. KKS102

**DOI:** 10.1002/2211-5463.12405

**Published:** 2019-01-30

**Authors:** Dayyabu Shehu, Zazali Alias

**Affiliations:** ^1^ Institute of Biological Sciences Faculty of Science University of Malaya Kuala Lumpur Malaysia; ^2^ Department of Biochemistry College of Health Sciences Bayero University Kano Nigeria

**Keywords:** *Acidovorax* sp. KKS102, bioremediation, dehalogenation, glutathione *S*‐transferase, site‐directed mutagenesis

## Abstract

A glutathione *S*‐transferase (GST) with a potential dehalogenation function against various organochlorine substrates was identified from a polychlorobiphenyl (PCB)‐degrading organism, *Acidovorax* sp. KKS102. A homolog of the gene *BphK* (biphenyl upper pathway K), named *BphK‐KKS*, was cloned, purified and biochemically characterized. Bioinformatic analysis indicated several conserved amino acids that participated in the catalytic activity of the enzyme, and site‐directed mutagenesis of these conserved amino acids revealed their importance in the enzyme's catalytic activity. The wild‐type and mutant (C10F, K107T and A180P) recombinant proteins displayed wider substrate specificity. The wild‐type recombinant GST reacted towards 1‐chloro‐2,4‐dinitrobenzene (CDNB), ethacrynic acid, hydrogen peroxide and cumene hydroperoxide. The mutated recombinant proteins, however, showed significant variation in specific activities towards the substrates. A combination of a molecular docking study and a chloride ion detection assay showed potential interaction with and a dechlorination function against 2‐, 3‐ and 4‐chlorobenzoates (metabolites generated during PCB biodegradation) in addition to some organochlorine pesticides (dichlorodiphenyltrichloroethane, endosulfan and permethrin). It was demonstrated that the behavior of the dechlorinating activities varied among the wild‐type and mutant recombinant proteins. Kinetic studies (using CDNB and glutathione) showed that the kinetic parameters *K*_m_, *V*_max_, *K*_cat_ and *K*_m_/*K*_cat_ were all affected by the mutations. While C10F and A180P mutants displayed an increase in GST activity and the dechlorination function of the enzyme, the K107T mutant displayed variable results, suggesting a functional role of Lys107 in determining substrate specificity of the enzyme. These results demonstrated that the enzyme should be valuable in the bioremediation of metabolites generated during PCB biodegradation.

**Abbreviations**
BphK: biphenyl upper pathway K
CDNB: 1‐chloro‐2,4‐dinitrobenzene
DDT: dichlorodiphenyltrichloroethane
GSH: reduced glutathione
GST: glutathione *S*‐transferase
PCB: polychlorobiphenyl
RMSD: root mean square deviation

Glutathione *S*‐transferases (GSTs) constitute a diverse class of enzymes that detoxify different classes of substrates by catalyzing their conjugation with the tripeptide reduced glutathione (GSH) thereby helping to increase their solubility and subsequent removal from the body 1, 2. GSTs are found in almost all living organisms, including plants, animals, fungi and bacteria 3. They are found in the cytosol, mitochondria and microsomal fraction and this forms one of the bases of their classification. They are classified as cytosolic, mitochondrial, microsomal and bacterial specific fosfomycin‐resistant proteins 2.

*Acidovorax* sp. KKS102 is a biphenyl/polychlorobiphenyl (PCB) degrading organism isolated from a soil near a refinery in Japan 4. Polychlorobiphenyls are persistent but industrially useful chemicals that were released many decades ago and still continue to pose a danger to humans, other animals and the environment. They are carcinogenic, endocrine‐disrupting chemicals and were found to have an effect on the liver and nervous system. Because of their recalcitrance, they are among the chemicals listed by the Stockholm Convention for eventual elimination by 2025.

*Acidovorax* sp. KKS102 shows promise for the biodegradation of several congeners of polychlorobiphenyls including those that are recalcitrant to other PCB degraders 5. The organism was found to always live in symbiotic relationship with a benzoic acid degrader, *Pseudomonas fluorescence* KKL101 6. While the duo were shown to completely mineralize biphenyls, the PCBs could not be completely metabolized and chlorobenzoates remained as one of the dead‐end metabolites 5. This presence of chlorobenzoates as dead‐end metabolites in PCB biodegradation presents a problem as they are toxic to the PCB degraders 7, 8. Typically, PCBs consist of at least 60 different congeners, and microorganisms were shown to act on only a fraction of these 9. This prompted research into various ways to optimize the activities of related enzymes in PCB biodegradation 10. However, most of the research on enzyme optimization was mainly directed toward biphenyl‐1,2‐dioxygenase, as this is the key enzyme that specifies the types of congeners to be degraded by a PCB degrader 9. The presence of dead‐end metabolites presents another problem as they halt the biodegradation process, and hence the need for research on how to deal with them 8.

The function of one enzyme located within the biphenyl upper pathway for biphenyl/PCB degradation and designated biphenyl upper pathway K (BphK), found in organisms such as *Burkholderia xenovorans* LB400, was initially obscure 11 but later shown to be a GST. Studies have shown that BphK had a dechlorination function against some toxic metabolites of polychlorobiphenyl degradation and some organochlorine pesticides 12, 13. However, it was discovered that the enzyme has limited substrate specificity and low catalytic activity 8. Furthermore, not all PCB degraders were found to contain the gene *BphK* located within the *bph* operon. This leads to the suggestion that studying other BphK homologs from biphenyl/PCB degrading organisms might lead to the identification of a GST having a better dechlorination function as well as wider substrate specificity against these toxic metabolites.

Various attempts have been made to improve the biodegradation capability of *Acidovorax* sp. KKS102 including the insertion of a constitutive promoter that enhances the overexpression of the *bph* genes and alleviation of toxic effects of biphenyl by degradation 5, 6. However, none of the studies focused on how to deal with the dead‐end metabolites created during the biodegradation process. *Acidovorax* sp. KKS102 was found to contain many putative GSTs even though none of them was found to be located within its *bph* operon. This research was aimed at identifying a suitable homolog from these GSTs and studying its biochemical properties with the aim of identifying a novel enzyme that could be employed to genetically engineer a strain with superior degradation capability or that could be used against the dead‐end metabolites of PCB biodegradation. Some organochlorine pesticides not previously determined in other studies were also employed as possible substrates for the GST.

## Materials and methods

### Organism

*Acidovorax* sp. KKS102 (JCM 17234) was obtained in freeze‐dried form from the Japan Collection of Microorganisms (JCM; Tsukuba, Japan). The organism was revived using Luria–Bertani (LB) broth according to the JCM's instructions.

### Chemicals

Unless otherwise stated, chemicals employed were of the highest grade obtainable. 2‐Chlorobenzoates, 3‐chlorobenzoates, 4‐chlorobenzoates, dichlorodiphenyltrichloroethane (DDT), endosulfan and permethrin were purchased from Merck Millipore, Burlington, MA, USA. 1‐Chloro‐2,4 dinitrobenzene (CDNB), ethacrynic acid, hydrogen peroxide, cumene hydroperoxide, GSH, NADPH and glutathione reductase were all purchased from Sigma‐Aldrich (St Louis, MO, USA). Molecular biology reagents were purchased from Thermofisher Scientific, Waltham, MA, USA. QuickChange lightning site‐directed mutagenesis kit was purchased from Agilent Technologies (Santa Clara, CA, USA).

### Bioinformatic analysis

The complete genome sequence of *Acidovorax* sp. KKS102 (accession no.: CP003872.1) deposited at the National Center for Biotechnology Information (NCBI) was used to identify putative GSTs in this organism. A separate search for *BphK* sequences from other organisms was also performed for comparative purposes. A sequence alignment study was carried out using clustalw2 14. Phylogenetic analysis was carried out using Molecular Evolutionary Genetic Analysis (mega) software version 6.0 15. The neighbor‐joining method was used to trace the evolutionary history of the GSTs 16. The Reltime method was used to calculate the divergence time for all the branch points 17.

### PCR amplification and cloning of wild‐type *BphK‐KKS* gene

Isolation of genomic DNA from *Acidovorax* sp. KKS102 was carried out using the PrepEase Genomic DNA Isolation Kit (Affymetrix Inc., Santa Clara, CA, USA) and was used for PCR amplification of the *BphK‐KKS* gene. The gene was successfully amplified using primers (forward 5′‐CACCATGAAGCTCTACTACGCCCCCGGT‐3′ and reverse 5′‐TCACGACAGCAACCCCTCAGCCCGCA‐3′). The PCR reaction was set using a three‐step PCR set‐up: (a) one cycle of initial denaturation at 98 °C for 10 s, (b) 30 cycles of denaturation at 98 °C for 1 s, 68.5 °C annealing for 5 s and 72 °C extension for 15 s per kb, and (c) one cycle of final extension at 72 °C for 10 min. The amplified products were purified and cloned into the pET101 D‐TOPO vector (Thermofisher Scientific). The successful clone was isolated and sequenced for further confirmation.

### Site‐directed mutagenesis

The method of Liu and Naismith 18 was adopted for site‐directed mutagenesis studies. The pair of primers used during the PCR reaction were as follows: for the C10P mutation, forward 5′‐CCGGTGCCT**T**CTCGCTCGCCGTCCA CATTGCCTTG‐3′ and reverse 5′‐GAGCGAG**A**AGGCACCGGGGGCGTAGTAGAGCTTCAT‐3′; for the K107T mutation, forward 5′‐CTGCACA**C**GGGCTTCAGCCCCTGGCTGTGGCAC‐3′ and reverse 5′‐GAAGC CC**G**TGTGCAGTTCGGTGCTGACGAAGGTG‐3′; and for the A180P, forward 5′‐ACCTGCAG**C**CCTGGATGGCACGCGTGGCGGCCCGCCC‐3′ and reverse 5′‐CCATCCAGG**G**CTGCAGGTGCGGGTAGGCAGTGAG CGGG‐3′. All mutations were confirmed by DNA sequencing. Purification and characterization of the mutants were carried out as described for the wild‐type.

### Protein expression and purification

Both the wild‐type and mutant recombinant *BphK‐KKS* were overexpressed using BL21^star^ (DE3) as follows. About 10 ng of recombinant plasmid was transformed into BL21^star^ (DE3) chemically competent cells. The transformation reaction was then transferred into 10 mL LB broth containing 100 μg·mL^−1^ ampicillin and was allowed to grow overnight at 200 r.p.m. and 37 °C. LB (500 mL) containing ampicillin (100 μg·mL^−1^) was then inoculated with the entire 10 mL from the overnight culture. The culture was grown at 37 °C with shaking (200 r.p.m.) until the attenuance at 600 nm reaches about 0.5. Isopropyl β‐d‐1‐thiogalactopyranoside was added to a final concentration of 1 mm to induce protein expression and the cells were grown for a further 3 h. The cells were harvested by centrifugation at 3000 ***g*** for 12 min at 4 °C. Lysis buffer containing protease inhibitor and lysozyme was added to the pellets to facilitate the breakdown of the cell wall. The cells were lysed by sonication and centrifuged at 5000 ***g*** for 60 min. The supernatant was collected for GST purification.

Chromatography was carried out using an ÄKTA Purifier FPLC (GE Healthcare, Chicago, IL, USA) equipped with unicorn software version 5.1 and a fraction collector (Frac900) for greater automation of the purification process. The clear crude enzyme was applied to a Glutathione Sepharose™ (GE Healthcare) High Performance column (GSTrapTM HP, 5 mL) and pre‐equilibrated with 25 mm sodium phosphate buffer pH 7.4 maintained at a flow rate of 0.5 mL·min^−1^. The column was thoroughly washed with buffer A to remove non‐specifically bound proteins. The bound GSTs were eluted with 10 mm glutathione in buffer A. The collected fractions with activities towards CDNB were pooled and concentrated using a centrifugal concentrator (Vivaspin 20 : 10 000; Sigma Aldrich).

### GST activity assay and determination of kinetic parameters

The specific activity of BphK‐KKS was determined using various standard GST substrates. BphK‐KKS was found to react with CDNB, ethacrynic acid, hydrogen peroxide and cumene hydroperoxide out of many standard GST substrates tested. The GST activity towards CDNB and ethacrynic acid was carried out as described by Habig *et al*. 19. For CDNB, the reaction mixture (3 mL) contained 50 mm sodium phosphate buffer (pH 6.5), 1 mm CDNB, 1 mm GSH, and a suitable amount of purified enzyme. The reaction was started by the addition of the substrate and the rate of the reaction was recorded following the increase in absorbance at 340 nm. A similar reaction was performed for ethacrynic acid except that the substrate CDNB was replaced with 0.2 mm ethacrynic acid and the conjugation reaction was monitored at 270 nm. The peroxidase activities of the enzyme towards hydrogen peroxide and cumene hydroperoxide were carried out according to the method described by Di Ilio *et al*. 20. Both substrates (hydrogen peroxide and cumene hydroperoxide) were determined using a coupled assay in which the reduction of oxidized glutathione was coupled to the oxidation of NADPH by glutathione reductase. The assay mixture contained 50 mm Tris/HCl buffer pH 7.2, 1 mm GSH, 2.5 mm NADPH, 6 μg glutathione reductase, final concentrations of 1.2 mm for cumene hydroperoxide and 0.25 mm for hydrogen peroxide, and appropriate amounts of enzymes. The reaction was initiated by the addition of the substrates and the rate of the reaction was recorded following the decrease in absorbance at 340 nm. The kinetic parameters for wild‐type and *BphK‐KKS* mutants were determined using CDNB and GSH as substrates. For CDNB, this was performed by varying the CDNB concentration (0.3–2.1 mm) while keeping the GSH concentration constant. For GSH, it was determined by keeping the concentration of the CDNB constant while varying the GSH concentration (0.2–1.8). All the measurements were carried out using a Cary 60 UV‐Visible spectrophotometer (Agilent Tecnologies). The data were analyzed using non‐linear regression analysis with prism 7 software (GraphPad Software Inc., La Jolla, CA, USA). Kinetic parameters (*K*_m_ and *V*_max_) were calculated from the graph while *K*_cat_ was calculated using the equation *K*_cat_ = *V*_max_/[E]_t_.

### Chloride ion detection assay

The activity of BphK‐KKS towards chlorobenzoates (2‐, 3‐ and 4‐chlorobenzoates) and selected pesticides such as DDT, permethrin and endosulfan was measured by its ability to release chloride ions from the substrates. Chloride ions released were measured according to the procedure described by McGuinness *et al*. 13. Briefly, 900 μL of purified GST was incubated with 50 μL of 10 mm of GSH and 50 μL of 10 mm substrates overnight for 16 h at 100 r.p.m. and 28 °C. The reaction was terminated by the addition of 20 μL of 5 m H_2_SO_4_; thereafter, 200 μL of 13 mm Hg(SCN)_2_ in 95% ethanol and 200 μL of 0.25 m Fe (NH_4_)(SO_4_)_2_.12H_2_O in 9 m HNO_3_ were then added to 0.6 mL of the reaction mixture. The absorbance of Fe(SCN)^2+^ produced was measured after 5 min at 450 nm. The concentration of the chloride ions released was determined from the known concentration of sodium chloride in the standard curve.

### Protein concentration

The Bradford assay was used to determine the protein concentration using bovine serum albumin as a standard 21.

### SDS/PAGE

SDS/PAGE was performed in 12% polyacrylamide gel at 120 V using the method described by Laemlli 22. The gels were stained with Coomassie blue. The stained gels were scanned with Image Scanner III (GE Healthcare), and visualized and analyzed with image master software.

### Statistical analysis

Graphing and statistical analysis were performed using originpro 8.5 software (OriginLab Corp., Northampton, MA, USA). The 95% level of significance (*P* < 0.05) was used as the criterion to determine whether the variances of the data were statistically significant. All the data are reported as means ± SD of three independent experiments using different enzyme preparations.

### Molecular docking studies

A molecular docking study was performed using autodock 4.2 software 23. However, due to the absence of the crystal structure of BphK‐KKS, a 3D structure of BphK‐KKS was used throughout the molecular docking studies. The 3D structure was built using swiss model server 24. The server uses a suitable template from the experimentally determined structure of a related family of protein to build the 3D structure of the target protein. The BphK‐KKS model was built using a crystal structure of BphK from *B. xenovorans* LB400 (PDB code: 2gdr.1). The protein shares 48% sequence similarity with BphK‐KKS. The quality of the built model was assessed using GMQE and QMEAN scoring functions (http://swissmodel.expasy.org/qmean/cgi/index.cgi ). The model showed very high quality as indicated by values of 0.79 and −1.62 on GMQE and QMEAN scoring functions, respectively. The structures of all the ligands under study (mol2 file) were constructed and optimized using a chemsketch software. The mol2 files were then used as input for open babel software to generate the protein data bank (PDB) file of all the ligands 25. The PDB files of both the protein and ligands were used as input files in autodock tools (adt). Polar hydrogen, Kollman charges and solvation parameters were added to the protein while rotatable bonds for the ligands were defined. The prepared files were saved in the pdbqt format. Because of the variability in the substrate binding site of GSTs, a blind docking analysis was set up using an autogrid size of 126, 126 and 126 for the *x*,* y* and *z* axes respectively. A total of 100 runs were made for each binding site using the Lamarckian genetic algorithm as a search engine. The parameters used in the autodocking process were as follows: GA population size = 100, maximum number of energy evaluations = 250 000. Default parameters were used for mutation, crossover and elitism. Cluster analysis was performed on the docked result using a root mean square deviation (rmsd) of 2 Å. The docked conformations were visualized using discovery studio software.

## Results

### Bioinformatic analysis

**Figure 1:**
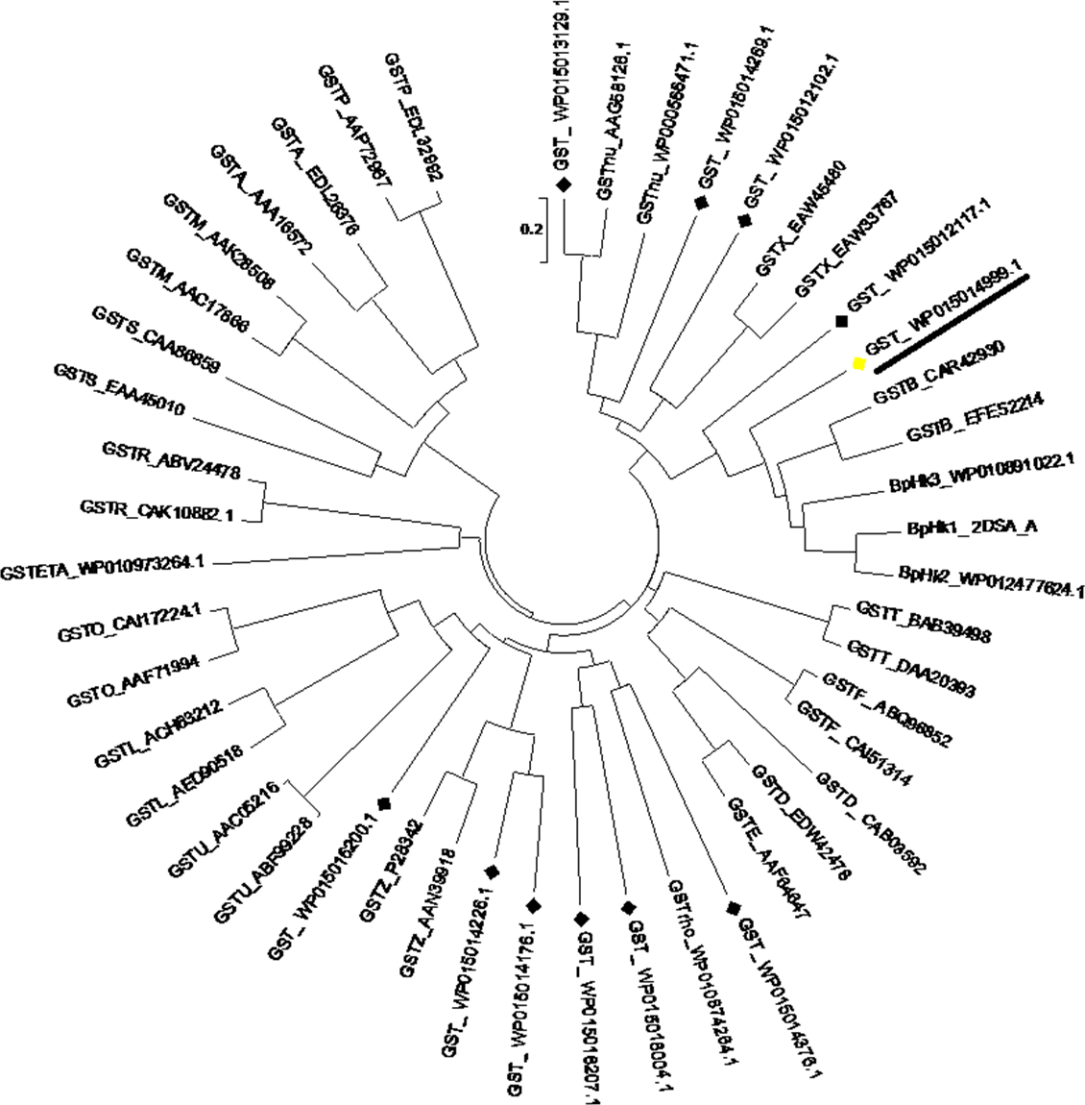
Phylogenetic tree indicating the relationship between 11 putative GSTs in *Acidovorax* sp. KKS102 with representative bacterial and eukaryotic GSTs. The phylogenetic tree was constructed using all known representative GST classes from both prokaryotic and eukaryotic organisms. A, alpha; B, beta; D, delta; E, epsilon; ETA, eta; F, phi; K, kappa; L, lambda; M, mu; nu, nu; O, omega; R, rho; S, sigma; T, theta; U, tau; P, pi; X, chi; Z, zeta. Numbers after the underscore are the NCBI accession numbers. Bold arrows represent *Acidovorax* sp. KKS102 putative GSTs. Underlining represents the BphK‐KKS from *Acidovorax* sp. KKS102 chosen for further studies.

**Figure 2:**
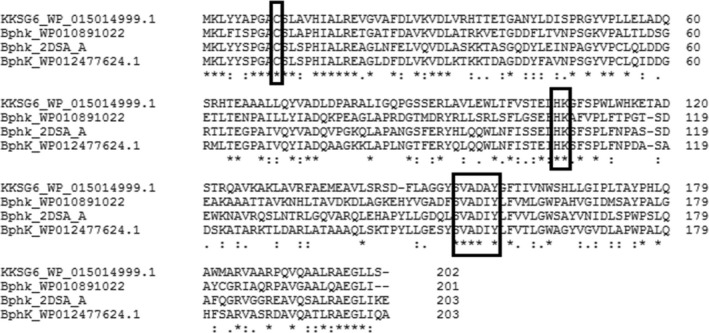
Multiple sequence alignment of BphK‐KKS (WP015014999.1) from *Acidovorax* sp. KKS102 and representative of BphK sequences from various PCB‐degrading bacteria. BphK (WP010891022.1) is from *Novosphingobium aromaticivorans*, BphK (2DSA_A) is from *Burkholderia xenovorans* LB400 and BphK (WP012477624.1) is from *Pseudomonas* sp. CT14. The numbers in parentheses are NCBI accession numbers. The boxed letters indicate the conserved amino acids known to affect the catalytic activity of the protein.

A bioinformatic analysis was carried out to identify a suitable BphK homolog from *Acidovorax* sp. KKS102 for further studies. *In silico* analysis of GSTs from *Acidovorax* sp. KKS102 showed that the organism contained 11 putative GSTs. Phylogenetic analysis using known GST classes revealed that two GSTs (GST_WP015012117.1 and GST_WP015014999.1) belonged to the same class as β‐class and BphK GSTs (Fig. 1). Further analysis using the sequence alignment studies revealed that GST_WP015014999.1 shared a greater percentage of sequence similarity (48%) with the *B. xenovorans* BphK LB 400 sequence compared with GST_WP015012117.1 (Fig. 2). Based on percentage similarity, GSTs that share 40% sequence similarity are included in the same class while those with less than 20–30% are assigned to a separate class 2. The protein also contained some conserved amino acids such as H106 and C10 as well as the conserved region of 152–158 (SVADIYL) which were reported to be essential in the catalytic activity of BphK LB400 26, 27. Thus, the gene was designated as *BphK‐KKS* and was selected for further studies. The gene was located at position 3622149–3622757 on the chromosome of *Acidovorax* sp. KKS102. It contained an open reading frame of 609 bp coding for a polypeptide of 202 amino acids with predicted molecular mass and theoretical isoelectric point (p*I*) of 22.14 kDa and 6.37 respectively.

### GST activity assay and effect of mutation of residues Cys10, Lys107 and Ala180 on the catalytic activity of BphK‐KKS

**Figure 3:**
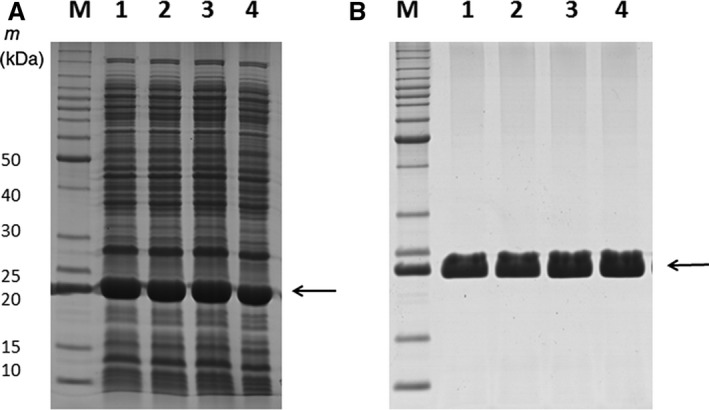
(A) SDS/PAGE analysis of wild‐type and mutant crude extracts of BphK‐KKS. M: benchmark (Thermofisher Scientific) molecular mass marker; lane 1: crude extract of BL21 (DE3) with wild‐type recombinant BphK‐KKS; lane 2: crude extract of C10F mutant; lane 3: crude extract of K107T mutant; lane 4: crude extract of A180P mutant. (B) SDS/PAGE analysis of purified wild‐type and mutant BphK‐KKS. M: bench mark (Thermofisher Scientific) molecular mass marker; lane 1: purified wild‐type KKSG6; lane 2: purified C10F mutant; lane 3: purified K107T mutant; lane 4: purified A180P mutant. Arrows in (A,B) indicate the purified recombinant BphK‐KKS.

In order to determine the substrate specificity BphK‐KKS, the full‐length sequence was successfully cloned into the pET101 D‐TOPO vector. Both the wild‐type and mutants were expressed and purified, and the purity was judged by SDS/PAGE (Fig. 3A,B). The specific activities of the mutants towards various standard GST substrates were measured and compared with the wild‐type (Table 1). The enzyme displayed wider substrate specificity using standard GST substrates compared with BphK GST from *B. xenovorans* LB400. BphK of *B. xenovorans* LB400 was reported to react with only CDNB 11, but BphK‐KKS was found to display activity towards CDNB, ethacrynic acid, hydrogen peroxide and cumene hydroperoxide. From the specific activity results (Table 1), BphK‐KKS displayed the highest activity with CDNB when compared with all other substrates. However, C10P and A180P displayed a 1.18‐ and 1.35‐fold, respectively, increase in catalytic activity of the protein while K107T mutant displayed a 3.47‐fold decrease in the catalytic activity towards CDNB. Surprisingly, while the K107T mutant had displayed a decrease in the catalytic activity of the protein towards CDNB, there was a 2.25‐fold increase in the catalytic activity of K107T mutant towards ethacrynic acid. C10F and A180P mutants both showed only a slight increase in the catalytic activity towards ethacrynic acid. The peroxidase activities of the wild‐type and all the mutants were not significantly affected except for the K107T mutant where there was 2.49‐ and 2.68‐fold decrease toward hydrogen peroxide and cumene hydroperoxide respectively.

**Table 1:** The results of specific activities of the recombinant wild‐type and mutant BphK‐KKS using various standard GST substrates. The results are means ± SD of three independent experiments. CuOOH, cumene hydroperoxide; EA, ethacrynic acid; CDNB, l‐Chloro‐2,4‐Dinitrobenzene; H_2_O_2_, Hydrogen peroxide

Enzyme	Specific activity (nmol·min^−1^·mg^−1^)			
	CDNB	EA	H_2_O_2_	CuOOH
WT	781.17 ± 13.86	96.95 ± 14.21	93.79 ± 16.79	88.07 ± 4.63
C10F	924.23 ± 9.91	121.60 ± 2.44	102.43 ± 3.95	98.48 ± 7.51
K107T	225.10 ± 5.57	217.90 ± 4.66	37.60 ± 5.11	32.83 ± 3.41
A180P	1056.30 ± 124.37	124.91 ± 15.61	86.23 ± 6.35	82.53 ± 6.60

**Table 2:** The Kinetic parameters of the recombinant wild‐type and mutant BphK‐KKS using CDNB and GSH as substrates. The results are means ± SD of three independent experiments

Enzyme	CDNB				GSH			
	*K*_m_ (mm)	*V*_max_ (mm ·min^−1^)	*K*_cat_ (min^−1^)	*K*_cat_/*K*_m_ (min^−1^·mm ^−1^)	*K*_m_ (mm)	*V*_max_ (mm ·min^−1^)	*K*_cat_ (min^−1^)	*K*_cat_/*K*_m_ (min^−1^·mm ^−1^)
WT	2.575 ± 0.140	0.124 ± 0.006	46.812 ± 5.621	19.295 ± 3.079	0.916 ± 0.138	0.032 ± 0.005	12.295 ± 3.079	20.345 ± 0.878
C10F	2.996 ± 0.070	0.142 ± 0.004	104.215 ± 7.966	34.749 ± 1.694	1.383 ± 0.179	0.068 ± 0.010	48.576 ± 3.192	35.619 ± 6.804
K107T	2.790 ± 0.142	0.078 ± 0.004	31.171 ± 1.084	11.661 ± 1.585	0.680 ± 0.037	0.030 ± 0.002	10.297 ± 1.533	15.224 ± 3.101
A180P	2.917 ± 0.128	0.150 ± 0.014	94.562 ± 6.813	32.516 ± 1.047	1.083 ± 0.001	0.065 ± 0.010	47.077 ± 1.627	43.394 ± 1.533

In order to investigate the behavior of the enzyme towards CDNB and GSH as substrates, the kinetic parameters were determined (Table 2). Determination of kinetic parameters of wild‐type and mutant BphK‐KKS using CDNB and GSH as substrates showed that mutation of Cys10 to Phe resulted in 1.16‐ and 1.51‐fold increase in the *K*_m_ toward CDNB and GSH, respectively, when compared with wild‐type. The increase in *K*_m_ signifies a decrease in the affinity of both the substrates toward *BphK‐KKS* and this resulted in 1.15 and 2.13‐fold increase in the V_max_ and 2.23 and 3.95‐fold increase in the K_cat_ of the enzyme toward CDNB and GSH, respectively. When Lys 107 was mutated to Thr, the *K*_m_ for the CDNB was found to slightly increase by 1.08‐fold while that of GSH was found to decrease by 1.35‐fold. Even though the mutation did not abrogate the activity of the enzyme toward CDNB, it did decrease the V_max_ by 1.56 and 1.07‐fold and K_cat_ by 1.50 and 1.19‐fold for CDNB and GSH respectively. When Ala180 was mutated to Pro in *BphK‐KKS*, there was 1.13‐ and 1.18‐fold increase in the *K*_m_ of the protein toward CDNB and GSH, respectively, when compared with wild‐type. The V_max_ increased by 1.21 and 2.03‐fold while the K_cat_ increased by 2.02 and 3.83‐fold for CDNB and GSH respectively.

### Analysis of binding interaction of BphK‐KKS and organochlorine substrates

**Figure 4:**
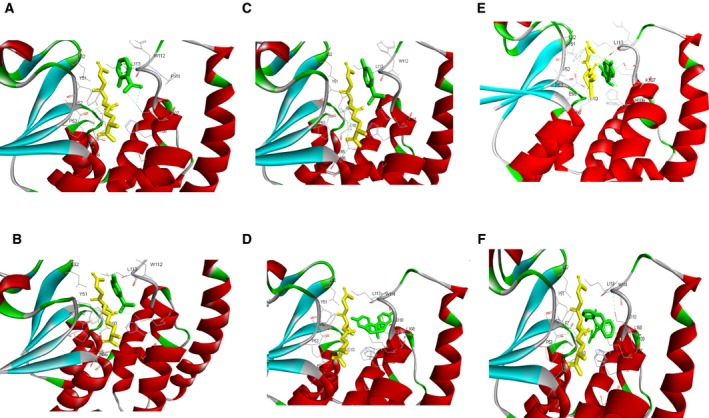
Predicted binding interaction of BphK‐KKS with various organochlorine substrates. The yellow ball and stick presentation indicates the co‐substrate GSH while the green ball and stick presentations represents the various organochlorine substrates, namely (A) 2‐chlorobenzoate, (B) 3‐chlorobenzoate, (C) 4‐chlorobenzoate, (D) DDT, (E) endosulfan, and (F) permethrin. The green dashed lines indicate hydrogen bonds between various amino acids in the binding pockets and the substrates.

A molecular docking study was performed in order to investigate the possibility of interaction and predict the binding site of various substrates of the BphK‐KKS. A blind docking was set up because of the high variability in the G site of GSTs. Analysis of the docking result with various substrates revealed the presence of several amino acids both in the G site and in the H site that were thought to play a role in the binding of the co‐substrate (GSH) and other chlorinated substrates. From the molecular docking result, the binding pocket was found to be occupied by amino acids A9, C10, L32, Y51, P53, V52, E65, S102, H106, K107, W112 and L113. Various interactive forces including hydrogen bonding and hydrophobic interactions were predicted to bind the substrates to the G and H sites. Cluster analysis of the docked result with 2‐chlorobenzoate using root mean square deviation (RMSD) tolerance of 2 Å revealed three different conformations. The lowest binding energy obtained was −5.78 kJ·mol^−1^, which occurred in the 14th run of the most populated cluster containing 46 members (Fig. 4A). With 3‐chlorobenzoate, the cluster analysis revealed four different conformations. The lowest minimum binding energy obtained was ‐6.01 kJ·mol^−1^, which occurred in the 24th run of the most populated cluster containing 59 members (Fig. 4B). 4‐Chlorobenzoate revealed three different conformations in the cluster analysis with the lowest minimum binding energy of −6.40 kJ·mol^−1^, which was obtained in the 98th run of the second most populated cluster containing 48 members (Fig. 4C). Analyses of hydrogen bond interactions with 2‐chlorobenzoate, 3‐chlorobenzoate and 4‐chlorobenzoate all predicted the presence of two hydrogen bonds between Lys107 and the ligand oxygen. This is in addition to the several hydrophobic interactions between the ligand atoms and the surrounding amino acids. In organochlorine pesticides, cluster analysis of docked conformations in DDT using RMSD tolerance of 2 Å revealed three different clusters. The lowest minimum binding energy obtained was −8.99 kJ·mol^−1^, which occurred in the 96th runs of the most populated cluster containing 76 members (Fig. 4D). Analysis of docked conformations in endosulfan showed seven different clusters. The lowest minimum binding energy obtained was −9.92 kJ·mol^−1^, which also occurred in the most populated cluster of 85 members (Fig. 4E). In contrast, cluster analysis of the docked conformation in permethrin showed a scattered distribution of the members with 48 different conformations. The lowest minimum binding energy of −10.70 kJ·mol^−1^ was obtained in the 13th run of a cluster containing seven members (Fig. 4F). The presence of several polar and hydrophobic amino acids in the binding cleft of BphK‐KKS might contribute to the stabilization of the complexes formed. However, only hydrophobic interactions seemed to be stabilizing BphK‐KKS with DDT and permethrin complexes. In contrast, both hydrogen bonding and hydrophobic interactions were found to be stabilizing the complex of BphK‐KKS with endosulfan. At least one hydrogen bonding was predicted between the hydrogen atom of Ser110 and the chlorine atom of the ligand, the hydrogen atom of Trp112 and the chlorine atom of the ligand and the hydrogen atom of Lys107 and one of the oxygen atoms from the ligand. The molecular docking result suggested the existence of interactions between BphK‐KKS and the organochlorine compounds, which were further investigated in the chloride ion detection assay.

### GST activity assay of wild‐type and mutant recombinant BphK‐KKS

**Figure 5:**
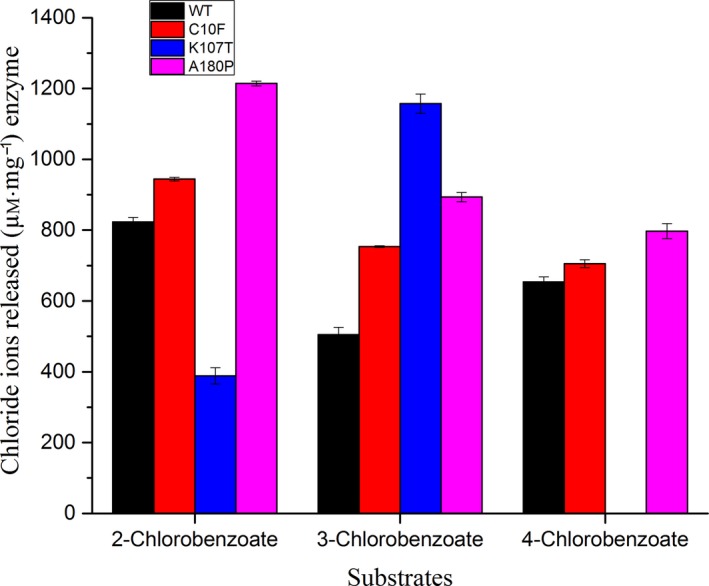
Dehalogenation of PCB degradation metabolites such as 2‐chlorobenzoates, 3‐chlorobenzoates and 4‐chlorobenzoates by BphK‐KKS. The results were expressed as amount of chloride ions released in μm·mg^−1^ of enzyme. The results are means ± SD of three independent experiments.

A chloride ion detection assay was used to measure the activity of purified wild‐type and the various mutants (C10F, K107T and A180P) of BphK‐KKS towards various organochlorine substrates. The result of the chloride ion detection assay using 2‐, 3‐ and 4‐chlorobenzoates as substrates is presented in (Fig. 5). In 2‐chlorobenzoate, the amount of chloride ion released by the wild‐type BphK‐KKS was found to be 823.53 ± 12.36 μm·mg^−1^ of purified protein. C10F and A180P showed a 1.15‐ and 1.47‐fold, respectively, increase in the dechlorination activity while K107T mutant displayed a 2.12‐fold decrease. The activity of wild‐type BphK‐KKS against 3‐chlorobenzoates was found to be 504.83 ± 20.86 μm·mg^−1^ of chloride ion released. In contrast, the three mutants, C10F, K107T and A180P, were found to respectively increase the dechlorination function by 1.49‐, 2.29‐ and 1.77‐fold. In 4‐chlorobenzoate, the wild‐type displayed 653.57 ± 14.24 μm·mg^−1^ of chloride ion released, while the C10F and A180P mutants were found to show an increase in the dechlorination function by 1.08‐ and 1.22‐fold, respectively. Surprisingly, the K107T mutant had completely lost its activity against 4‐chlorobenzoate; we examined the possible reason for this by molecular docking studies. From the molecular docking results, while the wild‐type BphK‐KKS showed a productive binding of 4‐chlorobenzoate substrate after 100 runs, the K107T mutant was found to show scattered and unproductive binding of 4‐chlorobenzoate (data not shown). This suggested that the residue Lys107 played a critical role in determining substrate specificity in this enzyme. This was further confirmed by the result just presented whereby a decrease in the activity was observed with respect to 2‐chlorobenzoate but an increase was observed with respect to 3‐chlorobenzoate in the K107T mutant.

**Figure 6:**
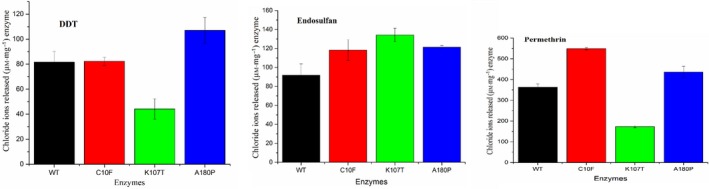
Dehalogenation of selected organochlorine pesticides, DDT, endosulfan and permethrin, by BphK‐KKS. The results are expressed as amount of chloride ions released in μm·mg^−1^ of enzyme. The results are means ± SD of three independent experiments.

For organochlorine pesticides, the GST activity of the wild‐type BphK‐KKS as measured by chloride ion detection assay using DDT as a substrate was 81.59 ± 8.54 μm·mg^−1^ of the purified protein (Fig. 6). A statistically significant 1.85‐fold reduction in the activity was observed in K107T mutant when compared with the wild‐type. However, in the case of C10F and Ala180P mutants, there was no statistically observable change in the activity of these mutants with DDT when compared with the wild‐type. In the case of endosulfan, the wild‐type BphK‐KKS showed 91.73 ± 11.95 μm·mg^−1^ protein of chloride ions released (Fig. 6). Unlike with DDT, the K107T mutant showed a statistically significant 1.46‐fold increase in activity toward endosulfan as compared with the wild‐type. However, while C10F did not show any statistically significant increase in catalytic activity, the A180P mutant showed a statistically significant 1.32‐fold increase in the catalytic activity of the protein when compared with the wild‐type. Permethrin, even though it structurally contains fewer chlorine atoms than DDT and endosulfan, showed severalfold greater GST activity when compared with DDT and endosulfan (Fig. 6). The K107T mutant showed a significant 2.11‐fold decrease in activity while C10F and A180P mutants showed a statistically significant 1.51‐fold and 1.20‐fold, respectively, increase in the dechlorination activity of the protein toward permethrin when compared with the wild‐type. This may be attributed to the low minimum binding energy in the docked conformation of permethrin (−10.70 kJ·mol^−1^) when compared with DDT (−8.99 kJ·mol^−1^) and endosulfan (−9.92 kJ·mol^−1^). The lowest minimum binding energy signifies better binding interaction and possible catalytic activity. The substrate‐dependent changes observed in the K107T mutant also suggest a probable function of the amino acid in determining substrate specificity in BphK‐KKS.

## Discussion

The present study demonstrated the presence of a BphK homolog in the putative GSTs of *Acidovorax* sp. KKS102. The enzyme displayed wider substrate specificity by reacting with ethacrynic acid in addition to CDNB unlike what was reported in BphK LB400, which reacts only with CDNB. Furthermore, the enzyme was found to possess peroxidase functions towards hydrogen peroxide and cumene hydroperoxide. Analysis of functional roles of some amino acids also revealed that they played a vital role in the catalytic activity of the protein. Several studies have shown that the catalytic mechanism of various classes of GSTs is mediated by their ability to lower the p*K*_a_ of the thiol group in the co‐substrate GSH thereby enhancing the nucleophilic attack on the diverse classes of electrophilic substrates. This function was found to be mediated by the cysteine residue at the N‐terminal domain of β‐class GSTs. Brennan *et al*. 26 showed that mutating Cys10 to Phe in *B. xenovorans* LB400 GST resulted in significant increase in the catalytic activity of the enzyme towards CDNB; the current study using BphK‐KKS showed that, in addition to increasing the activity of the enzyme toward various substrates, mutating Cys10 to Phe also affected all the kinetic parameters and increases the efficiency of catalysis of BphK‐KKS. The increase in the catalytic activity of the C10F mutant might be a result of the aromatic ring in the phenylalanine, which is absent in the cysteine residue. The phenylalanine was thought to form a stack with other aromatic rings in some proteins thereby increasing the catalytic activity of the protein 26. From the molecular docking results, Lys107 was found to be the only amino acid that formed hydrogen bonds with several substrates under study. Sequence alignment study using other BphK proteins also showed that Lys107 is a conserved amino acid across all the BphK amino acid sequences. In order to investigate the functional role of the amino acid in the catalytic activity of the protein, it was mutated to threonine. Lysine is a charged amino acid while threonine is a polar amino acid. Mutating Lys107 to Thr was found to give variable results for the catalytic activity of BphK‐KKS. While the specific activity of the K107T mutant toward CDNB and the peroxidase was found to decrease, the specific activity was found to significantly increase in the case of ethacrynic acid. This suggested that the residue played a very important role in determining substrate specificity in BphK‐KKS. Ala180 is not a strictly conserved amino acid in all the BphKs based on the sequence alignment study (Fig. 2). McGuiness *et al*. 13 reported that mutation of Ala180 to Pro resulted in an increase in the dehalogenation function of BphK LB400 towards some organochlorine pesticides and herbicides. However, mutating Ala180 to Pro in BphK‐KKS was found to increase the catalytic activity of the protein toward the substrates CDNB and ethacrynic acid. The mutation of Ala to Pro is thought to produce a kink in the protein structure thereby enhancing the catalytic activity of the protein 27.

BphK‐KKS also was found to display a dehalogenation function against chlorobenzoate substrates and some organochlorine pesticides. Of particular importance is the dehalogenation reaction observed in chlorobenzoate substrates (the dead‐end metabolites of PCB biodegradation). This will be useful in the bioremediation of these dead‐end metabolites as they were shown to be detrimental to some of the enzymes in the PCB degrading bacteria. Furthermore, dehalogenation of organochlorine pesticides will reduce their recalcitrance and exposes their carbon atom for further attack by other degradative enzymes. The complete loss of activity observed in the K107T mutant toward 4‐chlorobenzoate further suggested the importance of the residues in determining substrate specificity in BphK‐KKS. However, molecular docking results should be treated with caution as the minimum binding energy observed in organochlorine pesticides is much lower compared to what was observed in chlorobenzoate substrates; nevertheless, the amount of chloride ions released in chlorobenzoate is greater than what was observed in organochlorine pesticides. The minimum binding energy signifies better interaction between the enzyme and the substrate, and in theory, the compound with higher minimum binding energy should give a better dehalogenation function with regards to that substrate, which is not what was observed in organochlorine pesticides and chlorobenzoates. BphK‐KKS also displayed some peroxidase functions, which will be useful to the PCB degrading bacteria. Biodegradation of PCBs always results in the release of reactive oxygen species, which were shown to affect the efficiency of the biodegradation process. Addition of antioxidant to *B. xenovorans* LB400 was shown to improve the biodegradation of PCBs by the organism, since the BphK in LB400 does not possess peroxidase function 28. Some bacterial GSTs such as zeta, rho, beta and theta classes were shown to possessed peroxidase activities, which eventually helps in neutralizing the toxic effect of free radicals generated during metabolism 3, 29, 30. During PCB metabolism, the presence of GST with peroxidase function will eventually help in neutralizing the toxic effect that may be produced by the reactive oxygen species. This appeared to be another advantage possessed by BphK‐KKS as the peroxidase function of the enzyme will eventually help in neutralizing the free radicals generated during PCB biodegradation.

In conclusion, the results presented have demonstrated the potential of BphK‐KKS to be used in the bioremediation of metabolites of polychlorobiphenyl degradation and some organochlorine pesticides. Additional site‐directed mutagenesis on residue Lys107 will possibly unlocked additional properties of the enzyme that might be harnessed to engineer the GST in a way directed against a specific substrate. Furthermore, stability studies on various mutants will also widen our understanding about the possibility of their employment to accelerate the biodegradation process.

## Author contributions

ZA conceived and designed the project, DS performed the experiment, ZA analyzed the data, and DS wrote the paper, which was edited by ZA.

## Conflict of interest

The authors declare no conflict of interest.

## References

[1] Alias Z and Clark AG (2010) Adult *Drosophila melanogaster* glutathione S‐transferases: effects of acute treatment with methyl parathion. Pestic Biochem Physiol 98, 94–98.

[2] Allocati N , Federici L , Masulli M and Di Ilio C (2009) Glutathione transferases in bacteria. FEBS J 276, 58–75. 19016852 10.1111/j.1742-4658.2008.06743.x

[3] Sheehan D , Meade G , Foley V and Dowd C (2001) Structure, function and evolution of glutathione transferases: implications for classification of non‐mammalian members of an ancient enzyme superfamily. Biochem J 3, 1–16. 10.1042/0264-6021:3600001 PMC1222196 11695986

[4] Ohtsubo Y , Maruyama F , Mitsui H , Nagata Y and Tsuda M (2012) Complete genome sequence of *Acidovorax* sp. strain KKS102, a polychlorinated‐biphenyl degrader. J Bacteriol 194, 6970–6971. 23209225 10.1128/JB.01848-12 PMC3510582

[5] Ohtsubo Y , Shimura M , Delawary M , Kimbara K , Takagi M , Kudo T , Ohta A and Nagata Y (2003) Novel approach to the improvement of biphenyl and polychlorinated biphenyl degradation activity: promoter implantation by homologous recombination. Appl Environ Microbiol 69, 146–153. 12513989 10.1128/AEM.69.1.146-153.2003 PMC152473

[6] Kikuchi Y , Nagata Y , Ohtsubo Y , Koana T and Takagi M (1995) *Pseudomonas fluorescens* KKL101, a benzoic acid degrader in a mixed culture that degrades biphenyl and polychlorinated biphenyls. Biosci Biotechnol Biochem 59, 2303–2304. 8611753 10.1271/bbb.59.2303

[7] Adebusoye SA (2017) Biological degradation of 4‐chlorobenzoic acid by a PCB‐metabolizing bacterium through a pathway not involving (chloro) catechol. Biodegradation 28, 37–51. 27766437 10.1007/s10532-016-9776-3

[8] Seeger M and Pieper D (2010) Genetics of biphenyl biodegradation and co‐metabolism of PCBs In Handbook of Hydrocarbon and Lipid Microbiology (Timmis KN, McGenity TJ, van der Meer JR and de Lorenzo V, eds), pp. 1179–1199. Springer, Berlin, Heidelberg.

[9] Agulló L , Pieper DH and Seeger M (2017) Genetics and biochemistry of biphenyl and PCB biodegradation In Aerobic Utilization of Hydrocarbons, Oils and Lipids. Handbook of Hydrocarbon and Lipid Microbiology (Rojo F, ed), pp. 1–28. Springer, Cham.

[10] Pieper DH and Seeger M (2008) Bacterial metabolism of polychlorinated biphenyls. J Mol Microbiol Biotechnol 15, 121–138. 18685266 10.1159/000121325

[11] Bartels F , Backhaus S , Moore ER , Timmis KN and Hofer B (1999) Occurrence and expression of glutathione‐S‐transferase‐encoding bphK genes in *Burkholderia* sp. strain LB400 and other biphenyl‐utilizing bacteria. Microbiology 145, 2821–2834. 10537204 10.1099/00221287-145-10-2821

[12] Gilmartin N , Ryan D , Sherlock O and Dowling D (2003) BphK shows dechlorination activity against 4‐chlorobenzoate, an end product of bph‐promoted degradation of PCBs. FEMS Microbiol Lett 222, 251–255. 12770715 10.1016/S0378-1097(03)00309-4

[13] McGuinness M , Mazurkiewicz V , Brennan E and Dowling D (2007) Dechlorination of pesticides by a specific bacterial glutathione S‐transferase, BphKLB400: potential for bioremediation. Eng Life Sci 7, 611–615.

[14] Larkin MA , Blackshields G , Brown NP , Chenna R , McGettigan PA , McWilliam H , Valentin F , Wallace IM , Wilm A , Lopez R *et al* (2007) Clustal W and Clustal X version 2.0. Bioinformatics 23, 2947–2948. 17846036 10.1093/bioinformatics/btm404

[15] Tamura K , Stecher G , Peterson D , Filipski A and Kumar S (2013) MEGA6: molecular evolutionary genetics analysis version 6.0. Mol Biol Evol 30, 2725–2729. 24132122 10.1093/molbev/mst197 PMC3840312

[16] Saitou N and Nei M (1987) The neighbor‐joining method: a new method for reconstructing phylogenetic trees. Mol Biol Evol 4, 406–425. 3447015 10.1093/oxfordjournals.molbev.a040454

[17] Tamura K , Battistuzzi FU , Billing‐Ross P , Murillo O , Filipski A and Kumar S (2012) Estimating divergence times in large molecular phylogenies. Proc Natl Aca Sci USA 109, 19333–19338. 10.1073/pnas.1213199109 PMC3511068 23129628

[18] Liu H and Naismith JH (2008) An efficient one‐step site‐directed deletion, insertion, single and multiple‐site plasmid mutagenesis protocol. BMC Biotechnol 8, 91–96. 19055817 10.1186/1472-6750-8-91 PMC2629768

[19] Habig WH , Pabst MJ and Jakoby WB (1974) Glutathione S‐transferases the first enzymatic step in mercapturic acid formation. J Biol Chem 249, 7130–7139. 4436300

[20] Di Ilio C , Sacchetta P , Bello ML , Caccuri AM and Federici G (1986) Selenium independent glutathione peroxidase activity associated with cationic forms of glutathione transferase in human heart. J Mol Cell Cardiol 18, 983–991. 3783732 10.1016/s0022-2828(86)80012-8

[21] Bradford MM (1976) A rapid and sensitive method for the quantitation of microgram quantities of protein utilizing the principle of protein‐dye binding. Anal Biochem 72, 248–254. 942051 10.1016/0003-2697(76)90527-3

[22] Laemmli UK (1970) Cleavage of structural proteins during the assembly of the head of *bacteriophage* T4. Nature 227, 680–685. 5432063 10.1038/227680a0

[23] Goodsell DS , Morris GM and Olson AJ (1996) Automated docking of flexible ligands: applications of AutoDock. J Mol Recognit 9, 1–5. 8723313 10.1002/(sici)1099-1352(199601)9:1<1::aid-jmr241>3.0.co;2-6

[24] Bordoli L , Kiefer F , Arnold K , Benkert P , Battey J and Schwede T (2009) Protein structure homology modeling using SWISS‐MODEL workspace. Nature Prot 4, 1–13. 10.1038/nprot.2008.197 19131951

[25] O'Boyle NM , Banck M , James CA , Morley C , Vandermeersch T and Hutchison GR (2011) Open Babel: an open chemical toolbox. J Cheminformatics 3, 1. 10.1186/1758-2946-3-33 PMC3198950 21982300

[26] Brennan E , McGuinness M and Dowling DN (2009) Bioinformatic analysis and *in vitro* site‐directed mutagenesis of conserved amino acids in BphK LB400, a specific bacterial glutathione transferase. Int Biodeter Biodegrad 63, 928–932.

[27] Gilmartin N , Ryan D and Dowling DN (2005) Analysis of the C‐terminal domain of Burkholderia sp. strain LB400 BphK reveals a conserved motif that affects catalytic activity. FEMS Microbiol Lett 249, 23–30. 16006062 10.1016/j.femsle.2005.05.056

[28] Ponce BL , Latorre VK , González M and Seeger M (2011) Antioxidant compounds improved PCB‐degradation by *Burkholderia xenovorans* strain LB400. Enzyme Microb Tech 49, 509–516. 10.1016/j.enzmictec.2011.04.021 22142725

[29] Board GP , Baker TR , Chelvanayagam G and Jermiin SL (1997) Zeta, a novel class of glutathione transferases in a range of species from plants to humans. Biochem J 328, 929–935. 9396740 10.1042/bj3280929 PMC1219006

[30] Perito B , Allocati N , Casalone E , Masulli M , Dragani B , Polsinelli M and Carmine D (1996) Molecular cloning and overexpression of a glutathione transferase gene from *Proteus mirabilis* . Biochem J 318, 157–162. 8761466 10.1042/bj3180157 PMC1217602

